# Pulsed Electric Field Enhances Flavonoid Accumulation and Antioxidant Capacity in Chickpea Sprouts by Activating the Phenylpropanoid Pathway and Antioxidant System

**DOI:** 10.3390/foods15203750

**Published:** 2026-10-21

**Authors:** Fuhao Wang, Jiyuan Xue, Xiangli Ding, Jianhua Tang, Yongqi Yin

**Affiliations:** 1 College of Food Science and Engineering, Yangzhou University, Yangzhou 210095, China; 243603118@stu.yzu.edu.cn (F.W.); mz120232144@stu.yzu.edu.cn (J.X.); 2 College of Tourism and Culinary Science, Yangzhou University, Yangzhou 210095, China; dingxl@yzu.edu.cn

**Keywords:** chickpeas, flavonoids, pulsed electric field, response surface methodology, germination

## Abstract

Chickpea is an important legume crop, characterized by its high nutritional value and abundant physiologically active compounds. Pulsed electric field (PEF), as an emerging non-thermal physical processing technology, has demonstrated unique advantages in regulating seed germination and the accumulation of secondary metabolites. In this study, chickpeas were subjected to germination combined with PEF treatment to optimize germination conditions, and the physiological and metabolic changes, as well as flavonoid biosynthetic patterns in chickpea sprouts under conventional germination and PEF-assisted germination were systematically investigated. The results showed that under the optimal conditions (12 h soaking, PEF treatment at 30 kV applied voltage, 6 µs pulse width, and 500 Hz frequency for 27 min, followed by 72 h germination), the total flavonoid content in chickpea sprouts reached a peak of 3025.27 ± 21.11 µg/g DW. Optimized PEF conditions markedly activated the enzyme activities of the phenylpropanoid pathway and induced the upregulated expression of their corresponding genes. Meanwhile, the activities of antioxidant enzymes and the expression levels of related genes in PEF-treated chickpea sprouts were also markedly enhanced. PEF can promote flavonoid accumulation and improve antioxidant capacity in chickpea sprouts by activating the phenylpropanoid metabolic pathway and the antioxidant system. This study contributes to the development of nutritious foods based on chickpea.

## 1. Introduction

Chickpea (*Cicer arietinum* L.), an annual or perennial self-pollinating herb, belongs to the genus Cicer of the Fabaceae family. As the world’s third most widely cultivated edible legume, chickpea originated in West Asia and the Middle East. With a cultivation history of more than 7000 years, it ranks among the earliest domesticated legume crops for human beings [1]. As an important part of legume crops, chickpeas not only offer high cost-effectiveness and a wide range of uses, but also provide a balanced and comprehensive set of nutrients [2]. Featuring comprehensive and well-balanced nutritional profiles, chickpeas contain proteins, carbohydrates, lipids, vitamins, minerals, dietary fiber, and other nutrients [3]. They are also rich in flavonoids, polyphenols, and saponins, and are thus known as the king of legumes [4]. More importantly, chickpea matrices accumulate abundant secondary metabolites, especially flavonoids and polyphenolic compounds [5]. Its predominant flavonoid components were biochanin A and formononetin. Flavonoids are widely distributed plant secondary metabolites. As core functional substances, they participate in multiple biological processes, including growth and development regulation, stress defense, and inter-organism signal crosstalk, serving as essential material foundations for plants to adapt to environments and survive. Flavonoids from chickpea exerted multiple beneficial effects on human health [6], such as free radical scavenging and antioxidant activity, anti-inflammation and immunomodulation, as well as antiosteoporosis effects.

Nevertheless, conventional plant-derived foods commonly contain antinutritional factors, including phytic acid, tannins, and protease inhibitors, which have long restricted the full utilization of nutritional and functional bioactive components in chickpea. These antinutrients interfered with nutrient absorption and utilization efficiency in the human body [7]. In recent years, driven by the growing consumer demand for functional foods, developing processing technologies to increase flavonoid content and bioavailability of chickpea has become a key research focus. Several studies have demonstrated that germination treatment improved the digestibility of starch and protein, reduced the contents of antinutritional factors (phytic acid and tannins), and markedly elevated total flavonoid, isoflavone contents, and antioxidant capacity of chickpeas [8,9]. Our previous study found that germination treatment effectively promotes the accumulation of flavonoids in chickpea sprouts, enhances antioxidant capacity, improves protein and amino acid profiles, and reduces anti-nutritional factor levels, thereby enhancing the nutritional functionality and bioaccessibility of chickpea sprouts in a cultivar-specific manner [10]. However, the enrichment of bioactive substances solely by germination was limited. Pulsed electric field (PEF) is an efficient nonthermal physical method. Under appropriate electric field intensity, PEF induces reversible electroporation and moderate oxidative stress in plant cells [11], thereby facilitating the biosynthesis of bioactive secondary metabolites in germinated tissues. PEF treatment triggered defense responses in plants such as tomato [12], carrot [13], apple [14], potato [15], and blueberry [16], provoked reactive oxygen species (ROS) accumulation, activated stress-defense pathways, and promoted the synthesis and accumulation of functional bioactive substances, including phenolic acids, flavonoids, and carotenoids. By activating phenylalanine ammonia-lyase (PAL) activity in carrots [14], PEF enhanced the levels of phenols, chlorogenic acid, and hydroxybenzoic acid. Meanwhile, it raised chlorogenic acid levels in potatoes [15] and enhanced the accumulation of caffeic acid, ferulic acid, and cis-lycopene in tomatoes [12], strengthening plant antioxidant and stress-tolerance capacity. Galindo et al. [15] reported that PEF treatment triggered rapid ROS production in potatoes, induced crosslinking of cell wall hydroxyproline-rich glycoproteins, and substantially promoted chlorogenic acid biosynthesis. López-Gámez et al. [13] confirmed that PEF elevated the respiration rate of carrots, stimulated ethylene and acetaldehyde production, enhanced PAL activity, and facilitated the accumulation of phenolic acids and flavonoids [17]. Furthermore, PEF has proven effective for bioactive substance enrichment in apple [18], blueberry [16], and romaine lettuce [19]. Through electroporation-mediated increases in cell membrane permeability and higher antioxidant enzyme activities, PEF favored flavonoid accumulation. PEF treatment was therefore an effective strategy to promote flavonoid accumulation in plants.

González-Casado et al. [20] illustrated that PEF modulated secondary metabolite accumulation patterns by altering respiration rate and ethylene emission in tomato. Leong et al. [21] observed that the enrichment effects of PEF on phenolics and carotenoids varied greatly among carrot cultivars, indicating cultivar-dependent regulation. Song et al. [22] found that PEF markedly increased antioxidant enzyme activities in *Scutellaria baicalensis* seeds and mung bean sprouts, which efficiently scavenged excess reactive oxygen species. Bagarinao et al. [23] verified that PEF strongly activated the phenylpropanoid pathway, boosted PAL and Chalcone Synthase (CHS) activities, and increased total flavonoid and polyphenol contents by 25–40% in soybean and pea sprouts relative to untreated controls. Nevertheless, few studies had addressed the regulatory effects of PEF on physiological/biochemical characteristics, flavonoid synthase activities, and flavonoid metabolic accumulation in chickpea. The optimal PEF processing parameters for chickpea remained undetermined, and the underlying physiological mechanisms by which PEF governed flavonoid biosynthesis in chickpea were still unclear.

In this study, response surface methodology was adopted to optimize the PEF processing parameters. The changes induced by PEF treatment in total flavonoid content, total phenolic content, antioxidant enzyme activities, and flavonoid-metabolizing enzyme activities of chickpea were investigated. Additionally, the transcriptional changes in genes associated with the antioxidant system and flavonoid metabolism under PEF treatment were analyzed. Based on the aforementioned considerations, we hypothesize that appropriate PEF treatment can serve as a mild abiotic elicitor, activating the phenylpropanoid pathway and antioxidant system during chickpea germination, thereby facilitating the biosynthesis and accumulation of flavonoids in the sprouts. The objective of this study is to optimize the PEF treatment conditions using response surface methodology and to test this hypothesis at both the enzyme activity and gene expression levels. To the best of our knowledge, this is the first study to systematically optimize PEF processing parameters for flavonoid enrichment in germinated chickpeas by integrating single-factor experiments with response surface methodology. Moreover, unlike previous studies that have primarily focused on the effects of PEF treatment on the apparent physiological metabolism of plants, this study systematically evaluated the impact of optimized PEF treatment on the activities of phenylpropanoid pathway metabolic enzymes, the antioxidant system, and the expression of key genes during chickpea germination. By clarifying the regulatory effects at the physiological, enzymatic, and transcriptional levels, we aim to elucidate the physiological and transcriptional molecular mechanisms underlying PEF-induced flavonoid enrichment.

## 2. Materials and Methods

### 2.1. Experimental Design

The chickpea seeds of Pinying No. 1 used in this experiment were bred and provided by the Xinjiang Institute of Ecology and Geography, Chinese Academy of Sciences, in 2024, and were stored at −20 °C until use. Seeds were sterilized with a sodium hypochlorite solution. After sterilization, the chickpea seeds were thoroughly rinsed with deionized water until the rinse solution reached a neutral pH, followed by soaking at 25 °C for 12 h. Afterward, the seeds were placed on a tray and allowed to germinate at 25 °C, spraying 10 mL of water on them daily during the germination period. During germination, samples were randomly collected and immediately frozen in liquid nitrogen for subsequent analysis.

### 2.2. Optimization of PEF Treatment Conditions for Chickpeas

Chickpeas were incubated under germination conditions and subjected to PEF treatments with varied frequencies, durations, and voltages, and samples were harvested at different germination stages. Chickpeas (10 g) were evenly placed in a treatment chamber between a pair of horizontally parallel stainless steel electrode plates (25 × 16 cm) with a fixed distance of 2 cm. A PEF power supply (HD 35-5, Tianjin Huida Electronic Components Factory, Tianjin, China) was used with the following pulse parameters: monopolar square-wave pulses with a pulse width of 6 µs. The chickpeas did not contact the upper electrode plate. Sample temperature was measured immediately before and after treatment (25 ± 1 °C), with a temperature increase of less than 1 °C, confirming the non-thermal nature of the treatment process. Based on the single-factor tests that confirmed the optimal ranges of germination time, PEF frequency, treatment duration, and voltage, the germination conditions were further optimized to enhance the total flavonoid accumulation in chickpea sprouts. The detailed experimental design was as follows.

(1) PEF treatment stage selection. Chickpea seeds and sprouts were divided into multiple treatment groups, with untreated samples set as the control group (CK). The PEF treatment was performed at a fixed voltage of 15 kV, frequency of 500 Hz, pulse width of 6 µs, and duration of 10 min at five different germination stages, including the dry seed stage (Seed), 12 h of seed soaking (S-12), 12 h of germination (G-12), 24 h of germination (G-24), and 48 h of germination (G-48). All samples were harvested 3 days after germination to screen the optimal PEF treatment stage with the highest total flavonoid content.
(2) Optimization of germination time. On the basis of the optimal PEF treatment stage screened above, chickpea samples were treated with PEF at 15 kV voltage, 500 Hz frequency, 6 µs pulse width, and 10 min duration. Samples were collected at 12, 24, 36, 48, 60, 72, 84, and 96 h (including 12 h seed soaking) after germination. The total flavonoid content was determined to select the optimal germination time for flavonoid accumulation.
(3) Optimization of PEF treatment duration. After 12 h of seed soaking, chickpea germs were treated with PEF at a fixed voltage of 15 kV, pulse width of 6 µs, and frequency of 500 Hz, with treatment durations set as 0, 5, 10, 15, 20, 25, and 30 min. All sprout samples were harvested at 84 h of germination for total flavonoid determination.
(4) Optimization of PEF treatment voltage. Following 12 h of seed soaking, chickpea germs were subjected to PEF treatment at a constant frequency of 500 Hz, pulse width of 6 µs, and fixed duration of 25 min, with gradient voltages of 5, 10, 15, 20, 25, 30, and 35 kV. Samples were collected at 84 h of germination to detect total flavonoid content.
(5) Optimization of PEF treatment frequency. After 12 h of seed soaking, chickpea germs were treated with PEF at a fixed voltage of 25 kV, pulse width of 6 µs, and constant duration of 25 min, with treatment frequencies ranging from 200 Hz to 1000 Hz (200, 300, 400, 500, 600, 700, 800, 900, and 1000 Hz). All samples were harvested at 84 h of germination for the measurement of total flavonoid content. Detailed experimental parameters are listed in Supplementary Table S1. A Box-Behnken design with three factors at three levels was employed, comprising 17 experimental runs, including 5 center-point replicates used to estimate pure error for the lack-of-fit analysis. The experimental design and data analysis were performed using Design-Expert software (Version 13, Stat-Ease Inc., Minneapolis, MN, USA).

### 2.3. Determination of Total Flavonoids and Total Phenols Contents

Total flavonoid content was determined according to Huang et al. [24]. A total of 0.2 g of sprouts was ground with 5 mL of methanol. The homogenate was evaporated to dryness in a water bath, and this process was repeated three times to ensure the complete extraction of flavonoids from the sample. The resulting precipitate was dissolved in 3 mL of methanol and centrifuged at 8000× *g* for 10 min to collect the supernatant. 0.1 mL of the supernatant was transferred into a tube, and 2 mL of methanol along with 0.5 mL of 5% sodium nitrite were added. After standing for 5 min, 0.5 mL of 10% aluminum nitrate solution was added, and the mixture was shaken well and allowed to stand for another 5 min. Subsequently, 4 mL of 4% NaOH was added, and the mixture was incubated for 15 min. Finally, the absorbance was measured at 510 nm. This study used rutin as a standard to construct a standard curve. A rutin stock solution of 10 µg/mL was prepared in 80% (*v*/*v*) methanol and serially diluted to give working standards of 1, 2, 3, 4, 5, and 6 µg/mL. Each standard was treated exactly as the sample extracts, and the absorbance was recorded at 510 nm. The calibration curve was constructed by linear regression of absorbance against rutin concentration over the range of 1–6 µg/mL, giving the equation Y = 0.1306 X + 0.0125 (*R*^2^ = 0.9987). Sample extracts were diluted so that their absorbance fell within the linear range.

Total phenolic content was determined according to Mencin et al. [25]. Briefly, 0.2 g sprout was extracted with 5 mL of 50% (*v*/*v*) methanol for 15 min. The extract was then centrifuged at 10,000× *g* for 15 min to obtain the supernatant. One milliliter of the supernatant was taken, and 1.0 mL of 0.2 mM Folin–phenol reagent and 2.0 mL of 2% (*w*/*v*) sodium carbonate solution were added. The mixture was allowed to react for 2 h in the dark to prevent photodegradation. Measure the absorbance at 765 nm. Draw a standard curve using gallic acid as the standard sample. A gallic acid stock solution of 100 µg/mL was prepared in methanol and diluted to 10, 15, 20, 25, 30, and 35 µg/mL. Aliquots were mixed with Folin–Ciocalteu reagent and sodium carbonate solution as described above, and the absorbance was measured at 765 nm. The calibration curve was linear over the range of 0–35 µg/mL, with the regression equation Y = 0.0268 X + 0.007 (*R*^2^ = 0.9998). Sample extracts were diluted so that their absorbance fell within the linear range. The unit of total phenolic content is µg Gallic Acid Equivalent/g DW.

### 2.4. Determination of Antioxidant Capacity and ROS Levels

Antioxidant capacity was assessed in 80% (*v*/*v*) methanol extracts prepared from the frozen sprout powder; the clarified supernatant (8000× *g*, 10 min) was used throughout. 1,1-diphenyl-2-trinitrophenylhydrazine (DPPH) and 2,2′-azino-bis 3-ethylbenzothiazoline-6-sulfonic acid (ABTS) radical-scavenging activities were measured as described by Xue et al. [26]. The ferric ion reducing antioxidant power (FRAP) solution was prepared following Rumpf et al. [27]. MDA and H_2_O_2_ levels were quantified following Yin et al. [28]. The $O_{2}^{-\cdot }$ content was quantified based on the method described by Zhao et al. [29]. Collect the supernatant by centrifugation after mixing the sample with phosphate buffer solution. Mix the supernatant with hydroxylamine hydrochloride solution and incubate for 1 h. Subsequently, measure the absorbance at 530 nm. Use sodium nitrite as a standard to generate a standard curve for calculating the $O_{2}^{-\cdot }$ content in the sample.

### 2.5. Key Enzyme Activities in the Antioxidant System

Antioxidant enzyme activities were assayed according to Yin et al. [28]. After shaking the frozen sprout powder at low temperature in a sodium phosphate buffer (pH 7.0, 50 mM) to mix thoroughly, centrifuged (10,000× *g*, 4 °C, 15 min), and the supernatant was collected to measure antioxidant enzyme activity. The enzyme activity units of catalase (CAT), POD, and SOD are defined as absorbance changes of 0.02 per minute at 240 nm, 470 nm, and 560 nm, respectively. APX activity was measured according to Bin et al. [30]. All enzyme activities are expressed as units per gram of dry weight (U/g DW) of the sprout sample.

### 2.6. Metabolic Enzyme Activity Assay

Metabolic enzyme activities were quantified in accordance with Ma et al. [31]. Extract the sample using Tris-HCl solution (0.1 M, pH = 8.9) and prepare the supernatant after centrifugation (10,000× *g*, 4 °C, 15 min) for enzyme activity determination. Specifically, for PAL activity, take crude enzyme extract, add boric acid buffer and L-phenylalanine solution, react at 37 °C for 60 min, then add hydrochloric acid to terminate the reaction, and measure the absorbance at 290 nm. C4H activity: Take crude enzyme extract, add Tris-HCl and L-cinnamic acid, incubate at 37 °C for 15 min, and then add hydrochloric acid to terminate the reaction. Measure the absorbance at 340 nm. 4CL activity: Take crude enzyme extract and add Tris-HCl, 4-coumaric acid, CoA, and ATP. Incubate at 37 °C for 15 min, then add hydrochloric acid to terminate the reaction, and measure the absorbance at 333 nm. All assays used crude enzyme extracts without substrates as blank controls. All enzyme activities are expressed as units per gram of dry weight (U/g DW) of the sprout sample.

### 2.7. Analysis of Relative Gene Expression Levels

Total RNA was extracted using a reagent kit (RC411, Vazyme, Nanjiing, China), and cDNA was obtained by reverse transcription using the RT-PCR Master Mix Kit (RR036A, Takara Co., Ltd., Tokyo, Japan). Fluorescence quantitative PCR analysis was performed using the SYBR Green method. The relative transcription level was calculated using the 2^−∆∆Ct^ method [28]. The primer sequences used in this study are shown in Supplementary Table S2. The melting curves of all primers confirm the presence of a single melting peak with amplification specificity, with amplification efficiencies ranging from 96% to 104% and *R*^2^ > 0.99. Actin was used as the internal reference gene.

### 2.8. Statistical Analysis

All experiments were performed in three independent replicates and expressed as mean ± standard deviation. Statistical analysis was carried out using SPSS 22.0 software. The significance test of data was conducted using one-way analysis of variance (ANOVA) combined with Tukey’s multiple comparisons at the 0.05 level.

## 3. Results

### 3.1. Effects of PEF Treatment Stages on Total Flavonoid Content of Chickpea Sprouts

**Figure 1:**
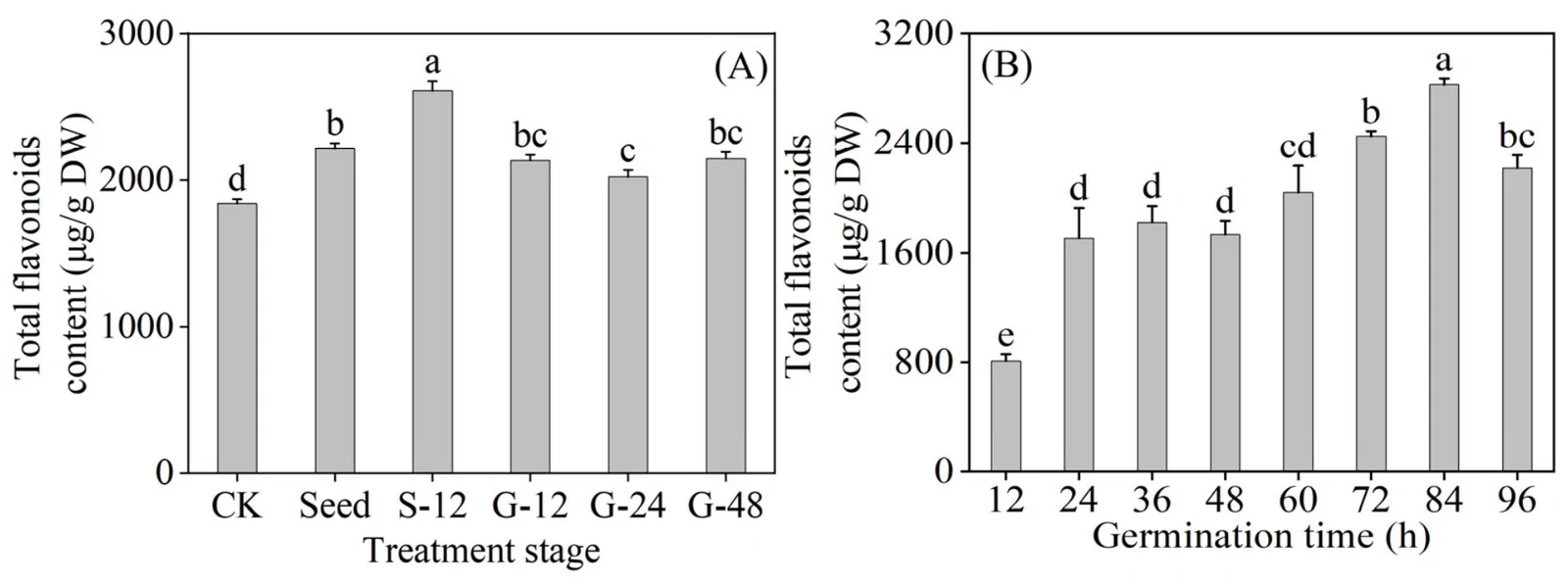
Effects of PEF at different treatment stages (**A**) and different germination times (**B**) on the flavonoid content in chickpea sprouts. Different letters indicate significant differences (*p* < 0.05). CK is the control group that does not undergo pulsed electric field treatment. The group at the seed stage is labeled as Seed, the group treated with soaking for 12 h is labeled as S-12, the group treated with 12 h germination treatment is labeled as G-12, the group treated with 24 h germination treatment is labeled as G-24, and the group treated with 48 h germination treatment is labeled as G-48.

**Figure 2:**
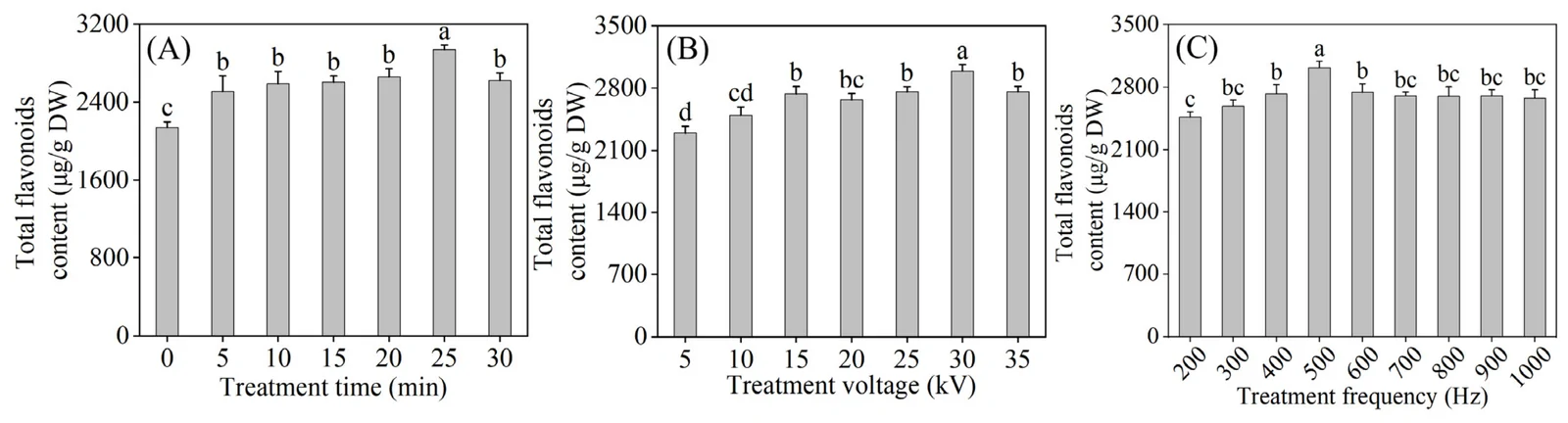
Effects of PEF with different treatment time (**A**), treatment voltage (**B**), and treatment frequency (**C**) on the total flavonoid content in chickpea sprouts. Different letters indicate significant differences (*p* < 0.05).

This study investigated the effects of PEF treatment stage and germination time on the total flavonoid accumulation of Pinying No. 1 chickpea sprouts. Firstly, chickpea seeds soaked for 12 h and germinated for 60 h were treated with PEF (15 kV, 500 Hz, 10 min) at five stages: dry seed, 12 h of soaking, 12 h, 24 h, and 48 h of germination. The total flavonoid content was determined at 72 h of germination. As shown in Figure 1A, the results showed that PEF treatment at 12 h of seed soaking produced the maximum flavonoid content of 2610.69 µg/g DW, which was higher than that of other groups. Based on the optimal treatment stage, a germination time gradient from 12 h to 96 h was further screened under identical PEF parameters. As shown in Figure 2B, the highest total flavonoid content of 2825.87 µg/g DW was observed at 84 h of germination with significant differences compared with other treatments (*p* < 0.05). Both PEF treatment stage and germination time significantly affected flavonoid accumulation in chickpea sprouts. The optimal basic conditions, namely PEF treatment at 12 h of seed soaking and germination for 84 h (including 12 h seed soaking), were determined for subsequent response surface optimization.

### 3.2. Effect of PEF Treatment Parameters on Total Flavonoid Content

Figure 2 showed that duration, voltage, and frequency of PEF treatment all exerted significant effects on the total flavonoid content of chickpea sprouts. Under the fixed optimal conditions of a 12 h seed soaking stage, 84 h germination time, and basic electric field parameters, the total flavonoid content first increased and then tended to stabilize with the variation of PEF treatment duration, voltage, and frequency. The maximum total flavonoid contents of 2942.97 µg/g DW, 2996.12 µg/g DW, and 3018.03 µg/g DW were obtained at a treatment duration of 25 min, a voltage of 30 kV, and a frequency of 500 Hz, respectively. In summary, the optimal single-factor parameters were determined, which provided a fundamental basis for subsequent response surface optimization experiments.

### 3.3. Optimization Results of RSM

A quadratic polynomial regression equation was established to describe the relationship between *Y* (total flavonoid content) and the three variables of A (PEF treatment duration), B (PEF treatment voltage), and C (PEF treatment frequency):

*Y* = 3017.24 + 88.39A − 20.52B + 103.47C + 91.21AB − 71.07AC − 92.07BC − 108.15A^2^ − 167.04B^2^ − 214.48C^2^

As shown in Table 1, the coefficient of determination *R*^2^ (*R*^2^ = 0.9917) was adopted to assess the fitting effect of the prediction model. The lack-of-fit F-value showed no statistical significance, which verified the reliability of the established model. Meanwhile, the *R*^2^_adj_ = 0.9810 indicated that the regression model reached a high significance level.

**Figure 3:**
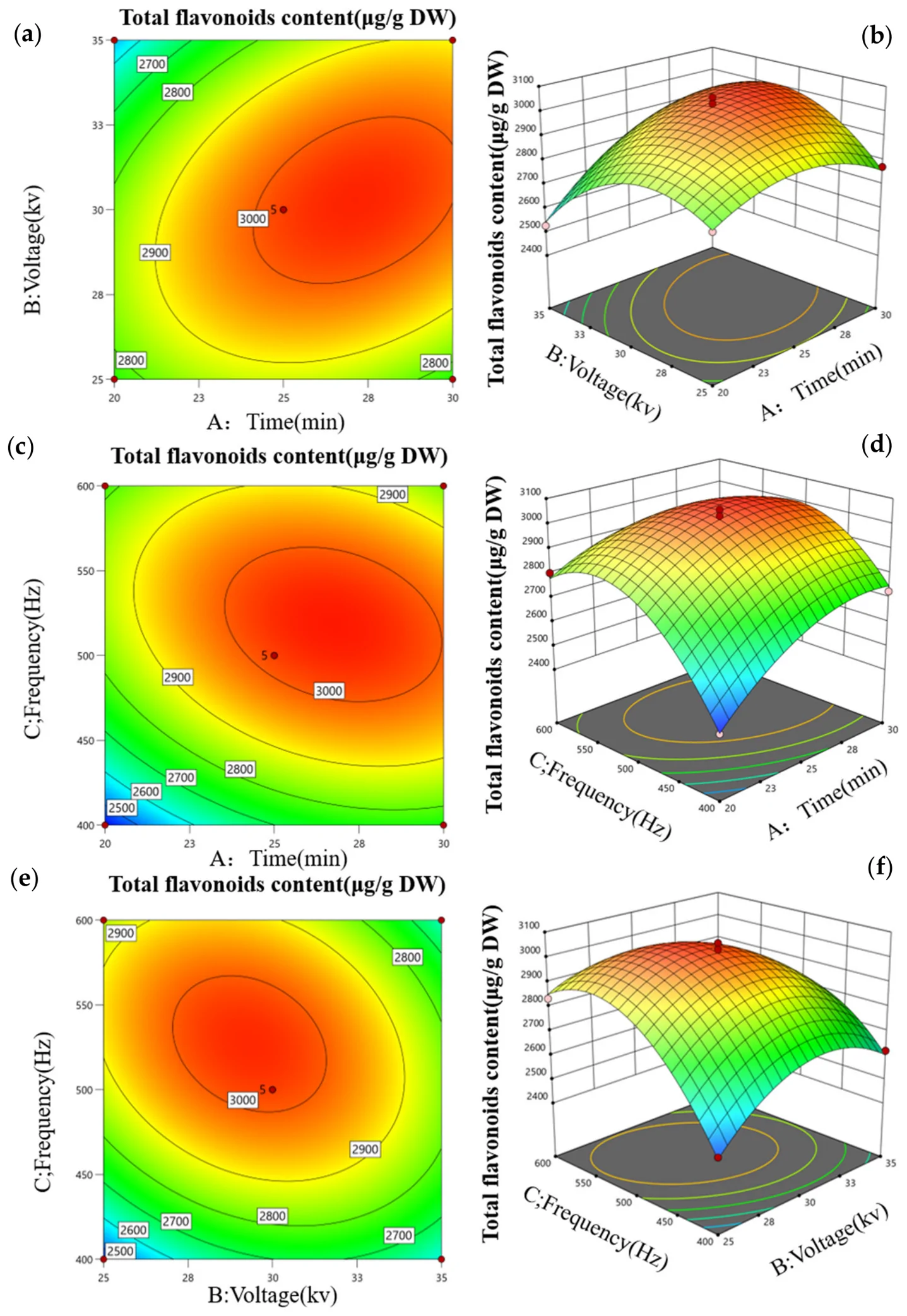
Response surface and contour plot of the interaction. The response surface plots show the interaction effects of each variable on the total flavonoid enrichment in chickpea sprouts, including the interaction between treatment time and treatment voltage, the interaction between treatment frequency and treatment time, and the interaction between treatment voltage and treatment frequency. Contour plots (**left**) and three-dimensional response surface plots (**right**) showing the interactive effects of treatment time (A) and voltage (B) at a fixed frequency of 500 Hz (**a**,**b**); treatment time (A) and frequency (C) at a fixed voltage of 30 kV (**c**,**d**); and voltage (B) and frequency (C) at a fixed treatment time of 25 min (**e**,**f**) on the total flavonoids content (µg/g DW) of chickpea sprouts. The color gradient from blue to red indicates increasing total flavonoids content, with blue representing the lowest values and red representing the highest values.

The interactive effects of each variable pair on total flavonoid enrichment in chickpea sprouts are presented in Figure 3. ANOVA results of the regression model confirmed that all these interactive effects reached an extremely significant level (*p* < 0.01).

To validate the optimum point predicted by the model (30 kV, 500 Hz, 27 min), we performed three independent validation experiments under the predicted optimal conditions. The mean observed total flavonoid content was 3025.27 ± 21.11 µg/g DW, which is in close agreement with the predicted value of 3037.62 µg/g DW, with a relative error of only 0.41%, confirming the adequacy and reliability of the model.

### 3.4. Effects of Optimized PEF Treatment on the Total Flavonoids and Total Phenolics Contents

**Figure 4:**
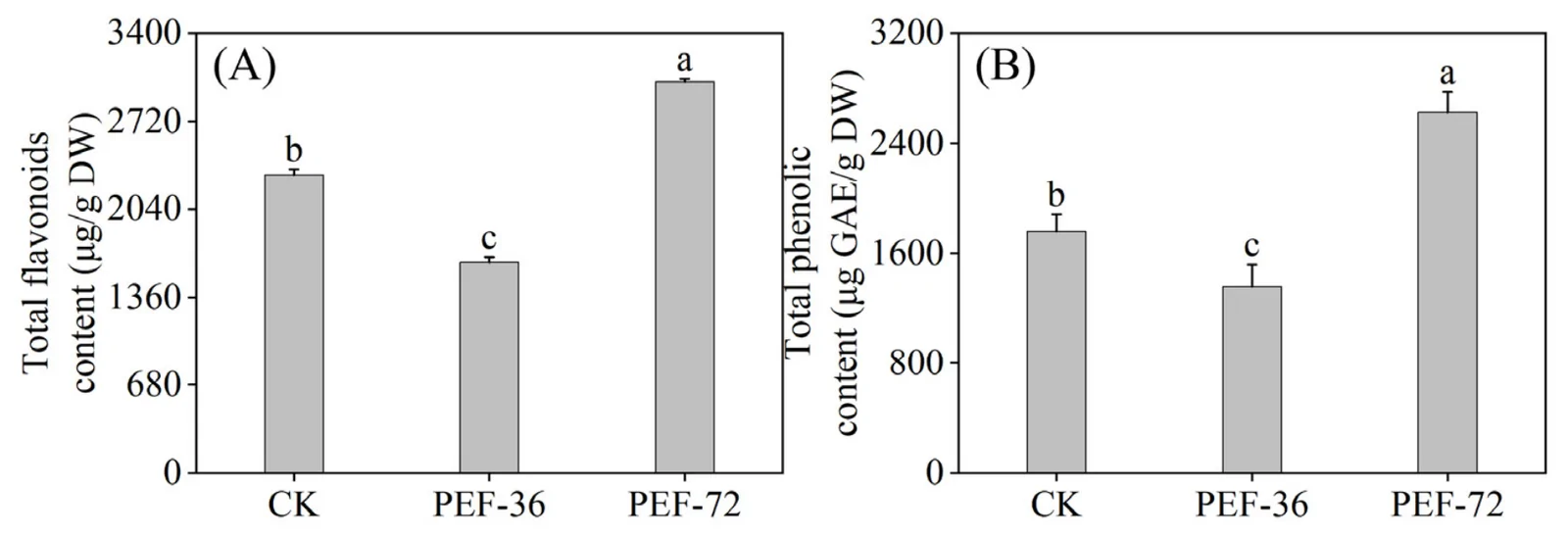
Effects of optimized PEF treatment on total flavonoids (**A**) and total phenolic content (**B**). CK—seeds soaked for 12 h without PEF treatment, followed by 72 h of germination (untreated control); PEF-36—seeds soaked for 12 h, subjected to PEF treatment (30 kV, 500 Hz, 6 µs pulse width, 27 min), followed by 36 h of germination; PEF-72—seeds receiving the same PEF treatment, followed by 72 h of germination. Different letters indicate significant differences (*p* < 0.05).

As shown in Figure 4, under optimized PEF treatment, the content of total flavonoids and total phenols in chickpeas continued to increase during germination. At 72 h of germination, the total flavonoid content in the PEF-treated group reached 3025.27 µg/g DW, which was significantly higher than that in the conventional germination group (*p* < 0.05), with a 1.31-fold increase. Meanwhile, the total phenol content reached 2626.71 µg/g DW, significantly higher than that in both the conventional germination group and the 36 h PEF-treated germination group (*p* < 0.05), which was 1.49 times that of the conventional germination group. The results indicated that PEF treatment under appropriate conditions could significantly promote the accumulation of total flavonoids and total phenols in chickpea sprouts (*p* < 0.05).

### 3.5. Effects of Optimized PEF Treatment on Antioxidant System

**Figure 5:**
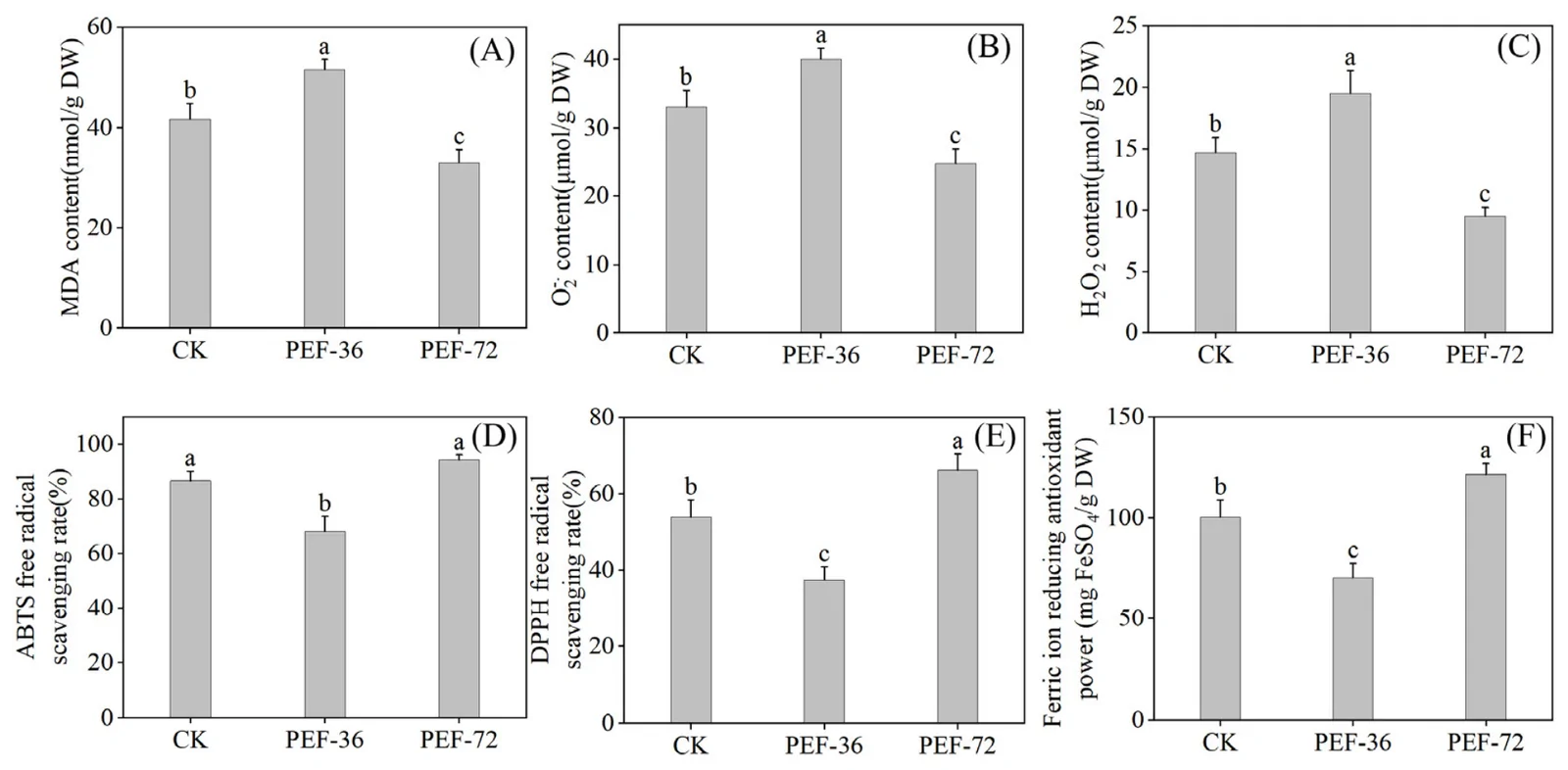
Effect of optimized PEF treatment on the levels of (**A**) MDA, (**B**) $O_{2}^{-}$, (**C**) H_2_O_2_, (**D**) ABTS, (**E**) DPPH, and (**F**) FRAP. Different letters indicate significant differences (*p* < 0.05). CK—seeds soaked for 12 h without PEF treatment, followed by 72 h of germination (untreated control); PEF-36—seeds soaked for 12 h, subjected to PEF treatment (30 kV, 500 Hz, 6 µs pulse width, 27 min), followed by 36 h of germination; PEF-72—seeds receiving the same PEF treatment, followed by 72 h of germination.

Figure 5 presents the MDA, $O_{2}^{-\cdot }$, and H_2_O_2_ contents in chickpea sprouts during germination with PEF treatment. After PEF treatment, the contents of all three substances decreased significantly with extended germination time (*p* < 0.05). Under optimized PEF treatment, the MDA and $O_{2}^{-\cdot }$ levels in 72 h germinated chickpea sprouts were 78.89% and 74.68% of the control, respectively, which were significantly lower than those in the control and PEF-36 groups (*p* < 0.05). The H_2_O_2_ content in the seedlings treated with PEF for 72 h was significantly lower than that in the control, at only 64.56% (*p* < 0.05). The results indicated that PEF treatment under appropriate conditions could significantly reduce the contents of MDA, $O_{2}^{-\cdot }$ and H_2_O_2_ in chickpea sprouts (*p* < 0.05).

### 3.6. Effects of Optimized PEF Treatment on Antioxidant Enzyme Activities

**Figure 6:**
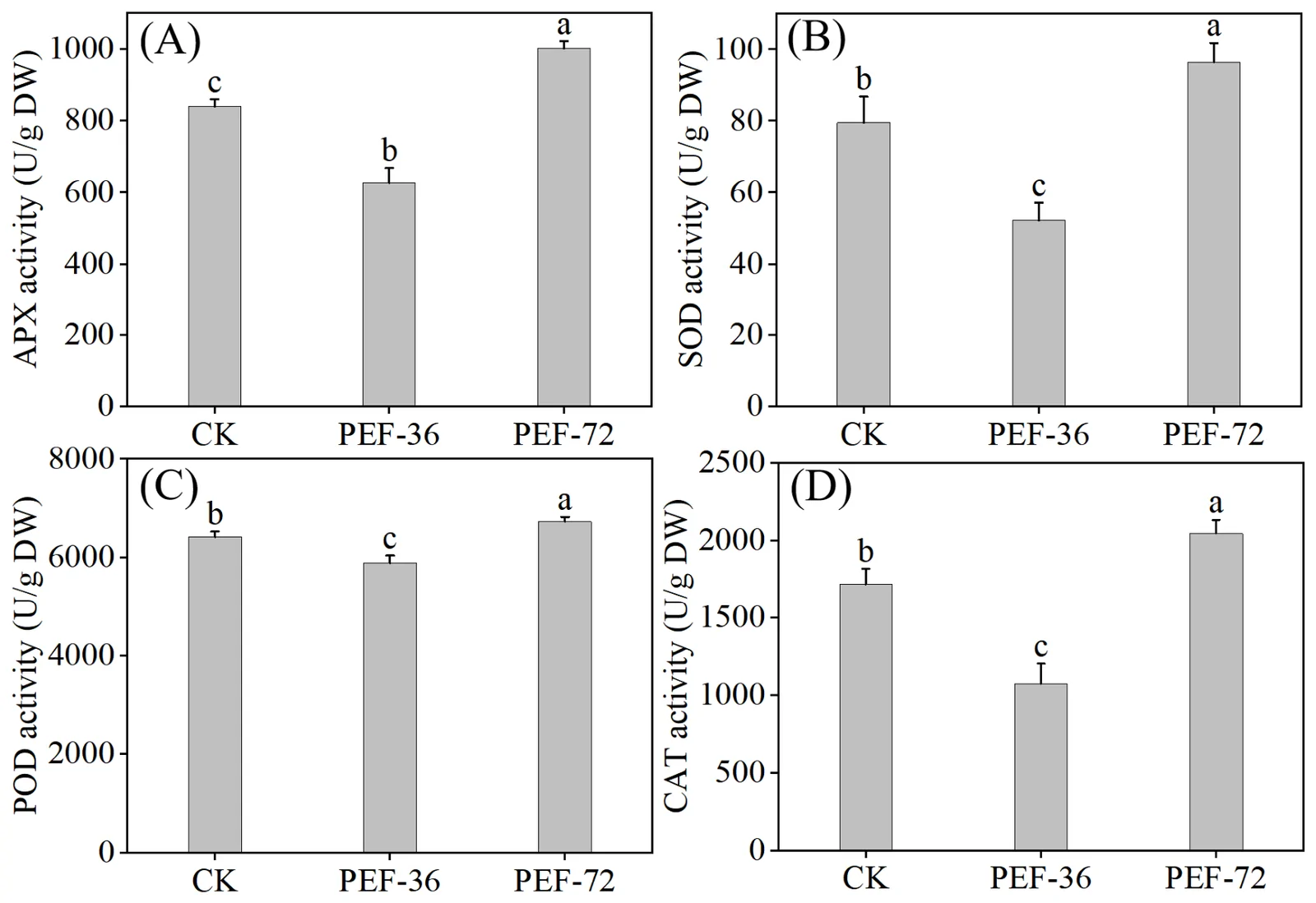
Effects of optimized PEF treatment on the activity of (**A**) APX, (**B**) SOD, (**C**) POD, and (**D**) CAT. Different letters indicate significant differences (*p* < 0.05). CK—seeds soaked for 12 h without PEF treatment, followed by 72 h of germination (untreated control); PEF-36—seeds soaked for 12 h, subjected to PEF treatment (30 kV, 500 Hz, 6 µs pulse width, 27 min), followed by 36 h of germination; PEF-72—seeds receiving the same PEF treatment, followed by 72 h of germination.

Figure 6 illustrates the activity changes of antioxidant enzymes in chickpea sprouts under PEF treatment. After PEF treatment, all enzyme activities exhibited an upward trend with extended germination time. At 72 h of germination, the activities of APX, SOD, POD, and CAT in the PEF-treated sprouts were markedly higher than those in the control group, which were 1.19, 1.21, 1.08, and 1.19 times those of the control group, respectively; the POD activity reached 6726.08 U/g DW. Meanwhile, the activities of APX and POD at 72 h were also significantly higher than those at 36 h after PEF treatment. The results demonstrated that PEF treatment under appropriate conditions could significantly enhance the antioxidant enzyme activities in chickpea sprouts (*p* < 0.05).

### 3.7. Effects of Optimized PEF Treatment on the Flavonoid Metabolic Enzyme Activities

**Figure 7:**
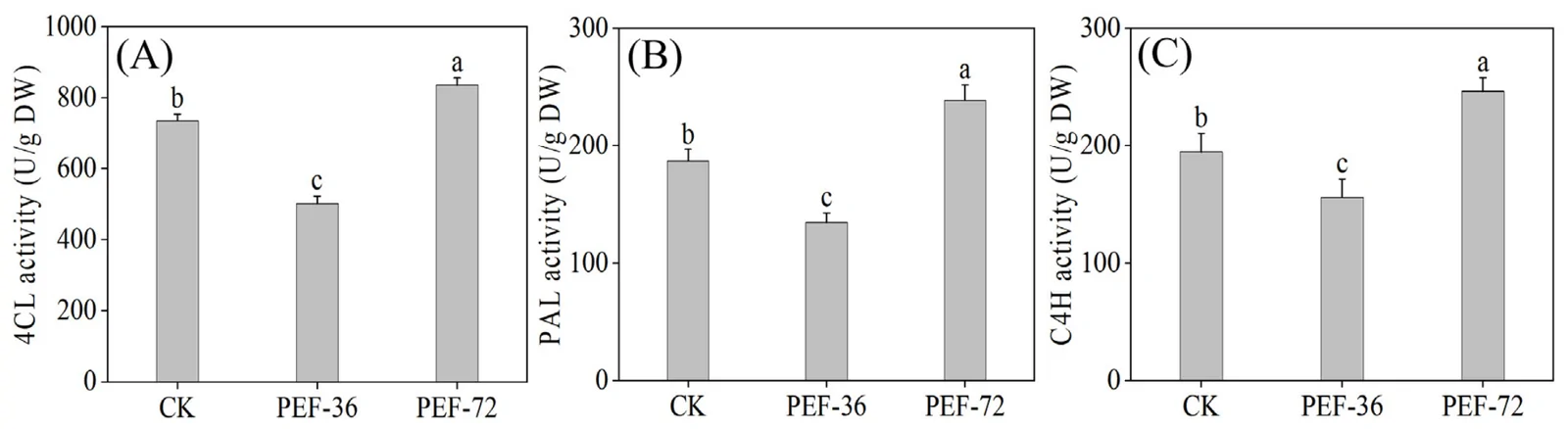
Effects of optimized PEF treatment on the activity of 4CL (**A**), PAL (**B**), and C4H (**C**). Different lowercase letters indicate significant differences (*p* < 0.05). CK—seeds soaked for 12 h without PEF treatment, followed by 72 h of germination (untreated control); PEF-36—seeds soaked for 12 h, subjected to PEF treatment (30 kV, 500 Hz, 6 µs pulse width, 27 min), followed by 36 h of germination; PEF-72—seeds receiving the same PEF treatment, followed by 72 h of germination.

After PEF treatment, the activities of three enzymes increased continuously with extended germination time (Figure 7). The activities of 4CL, PAL, and C4H in the PEF-72 treated group were significantly higher than those in the CK (*p* < 0.05), which were 1.14, 1.28, and 1.27 times those of the CK, respectively. Meanwhile, the 4CL and C4H activities at 72 h were also significantly higher than those at 36 h after PEF treatment. These results suggested that optimized PEF treatment could markedly enhance the activities of three enzymes in chickpea sprouts, thus effectively improving the flavonoid biosynthesis capacity.

### 3.8. Effect of Optimized PEF Treatment on Relative Expression Levels of Antioxidant Enzyme-Related Genes

**Figure 8:**
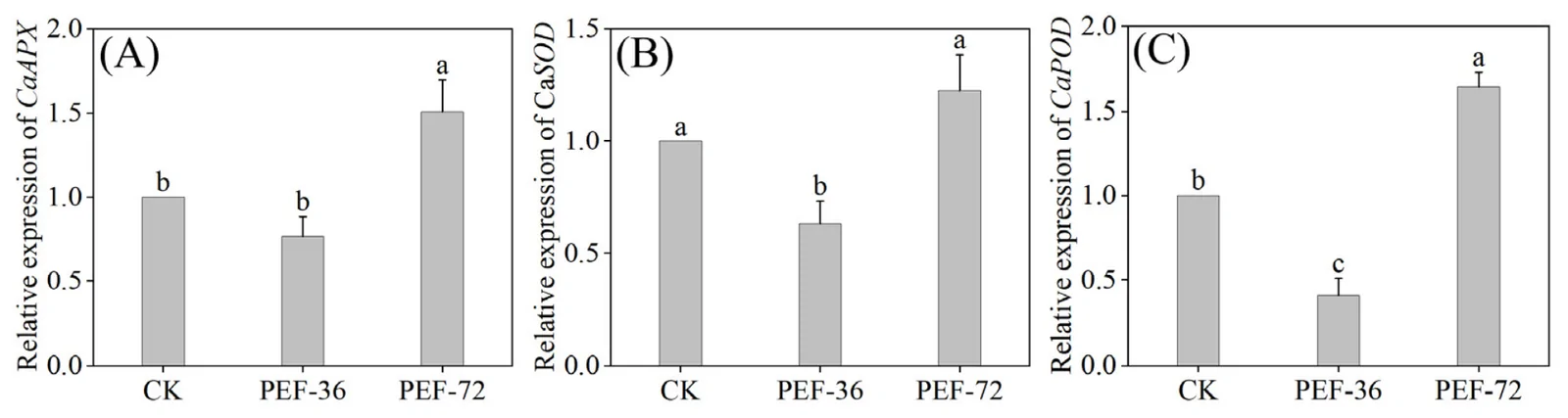
Effects of optimized PEF treatment on relative expression levels *of CaAPX* (**A**), *CaPOD* (**B**), and *CaSOD* (**C**). Different letters indicate significant differences (*p* < 0.05). CK—seeds soaked for 12 h without PEF treatment, followed by 72 h of germination (untreated control); PEF-36—seeds soaked for 12 h, subjected to PEF treatment (30 kV, 500 Hz, 6 µs pulse width, 27 min), followed by 36 h of germination; PEF-72—seeds receiving the same PEF treatment, followed by 72 h of germination.

Following PEF treatment, the relative expression of all antioxidant enzyme genes increased with germination time, and the expression levels at 72 h of germination were significantly higher than those at 36 h (Figure 8). At 72 h of germination, the relative expression levels of *CaAPX* and *CaPOD* in the PEF-treated group were significantly higher than those in the untreated control group (*p* < 0.05), which were 1.51-fold and 1.64-fold higher than the control, respectively. However, no significant difference was observed in the relative expression of *CaSOD* between the PEF-treated group and the control group. The results indicated that optimized PEF treatment could upregulate the relative expression of antioxidant enzyme genes in chickpea sprouts.

### 3.9. Effect of Optimized PEF Treatment on Relative Expression Levels of Flavonoid Biosynthetic Enzyme Genes

**Figure 9:**
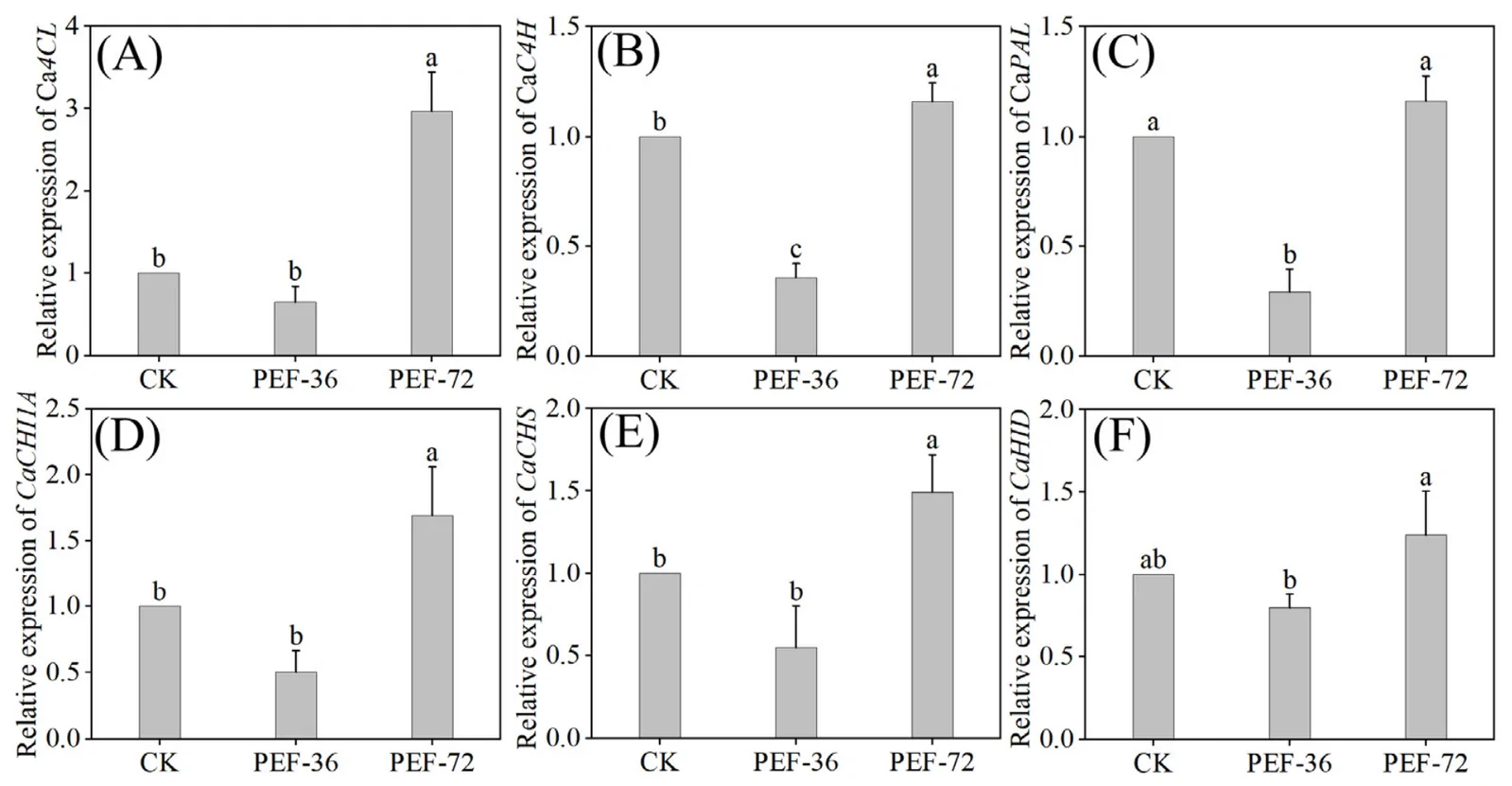
Effects of optimized PEF treatment on relative expression levels *of Ca4CL* (**A**), *CaC4H* (**B**), *CaPAL* (**C**), *CaCHI1A* (**D**), *CaCHS* (**E**), and *CaHID* (**F**). Different lowercase letters indicate significant differences (*p* < 0.05). CK—seeds soaked for 12 h without PEF treatment, followed by 72 h of germination (untreated control); PEF-36—seeds soaked for 12 h, subjected to PEF treatment (30 kV, 500 Hz, 6 µs pulse width, 27 min), followed by 36 h of germination; PEF-72—seeds receiving the same PEF treatment, followed by 72 h of germination.

Figure 9 illustrates the changes in relative expression levels of key genes in the phenylpropanoid metabolic pathway in chickpea sprouts under PEF treatment. After PEF treatment, the relative expression of all target genes increased with prolonged germination time, and the expression levels at 72 h of germination were significantly higher than those at 36 h. At 72 h of germination, the expression levels of *Ca4CL*, *CaC4H*, *CaCHI1A* and *CaCHS* in the PEF-treated group were significantly higher than those in CK (*p* < 0.05), which were 2.97-, 1.16-, 1.69- and 1.49-fold higher than the control, respectively. However, no significant differences were observed in the relative expression of *CaPAL* and *CaHID* between the PEF-treated group and the control group. The results indicated that PEF treatment could significantly upregulate the relative expression of key genes involved in the phenylpropanoid metabolic pathway related to flavonoid biosynthesis in chickpea sprouts (*p* < 0.05).

## 4. Discussion

As an emerging non-thermal physical processing strategy, PEF technology has gained extensive attention in food processing due to its low energy input, short processing duration, and well-preserved heat-sensitive bioactive components. For seed germination systems, precise regulation of electric field parameters (voltage, frequency, and treatment time) is a critical determinant of final treatment effects, as parameter shifts may trigger completely different cellular responses, from metabolic activation to irreversible damage. In the process optimization stage of this study, response surface results showed that moderate PEF treatment (30 kV, 500 Hz, 27 min) could significantly promote total flavonoid accumulation in chickpeas during germination, reaching a maximum of 3037.62 µg/g DW (Predicted value), whereas excessive voltage or prolonged treatment time would instead lead to a decline in flavonoid content. During the construction of the response surface model, although Factor B (voltage) was not statistically significant in the ANOVA (*p* = 0.0738), its quadratic term B^2^ (*p* < 0.0001) and interaction terms AB (*p* = 0.0003) and BC (*p* < 0.00003) were all highly significant. Therefore, we chose to retain the corresponding linear term B to preserve the hierarchical structure and interpretability of the quadratic polynomial model. Based on the experimental results, the combination of a non-significant linear term and a highly significant quadratic term suggests that Factor B (voltage) primarily influences flavonoid accumulation in a nonlinear, threshold-like manner rather than through a simple monotonic effect. Within the tested range, increasing the voltage to 30 kV significantly enhanced flavonoid accumulation, whereas a further increase to 35 kV resulted in a decline—a trend consistent with the transition from reversible electroporation to irreversible membrane damage. Previous studies have shown that the increased membrane permeability caused by electroporation during PEF treatment can accelerate physiological metabolism in the early germination stage [17]. Meanwhile, as an abiotic stress signal, PEF may trigger defense responses and activate secondary metabolic pathways in plants [32]. However, excessive PEF treatment can also inhibit plant physiological metabolism and the accumulation of flavonoids [33]. Therefore, the electric field parameters determined by response surface optimization in this study strike a balance between stress induction and cell viability and can effectively promote chickpea germination and total flavonoid enrichment. However, it must be acknowledged that the current study did not directly measure electrical conductivity or perform microscopic observations. Future studies should further provide robust support for PEF-induced changes in reversible electroporation and membrane micropore formation through such direct evidence.

Under normal physiological conditions, ROS such as superoxide anion and hydrogen peroxide are maintained at low steady-state levels by the endogenous antioxidant system. When plants suffer from PEF stress, excessive ROS production breaks this redox balance and induces oxidative damage. In this experiment, no massive accumulation of superoxide anion and H_2_O_2_ was observed in the 72 h PEF-treated group; instead, their contents were significantly lower than those in the untreated conventional germination group, accompanied by a parallel significant decrease in MDA content, a typical biomarker of membrane lipid peroxidation. This indicated that the antioxidant defense system of sprouts was successfully activated under optimized PEF treatment to cope with mild oxidative stress and maintain intracellular redox homeostasis [34]. The results of enzymatic activity and relative gene expression further revealed the internal mechanism. After PEF treatment, the activities of Ascorbate Peroxidase (APX), CAT, POD, and SOD in sprouts were all significantly increased, constituting a coordinated ROS scavenging network. At the transcriptional level, *CaAPX* and *CaPOD* genes were significantly upregulated, providing transcriptional support for enhanced antioxidant enzyme synthesis [35]. Furthermore, SOD activity in the 72 h PEF-treated group was significantly higher than that in the control group, but no significant difference was observed in *CaSOD* relative expression between the two groups. This discrepancy may result from the spatiotemporal dynamic lag between transcriptional and translational levels during plant stress response [36,37]. Gene transcription usually occurs rapidly at the early stage of stress exposure and peaks within hours, followed by rapid mRNA degradation to maintain transcriptional homeostasis [38]. By contrast, functional proteins translated from mRNA generally possess high structural stability and long half-lives once correctly folded in organelles [39]. Therefore, at 72 h of germination, *CaSOD* transcription has subsided, while SOD proteins synthesized at the early stage remain functionally active and continue to exert antioxidant effects. In addition to the enzymatic system, plants also build a non-enzymatic antioxidant barrier by synthesizing phenolic hydroxyl-rich secondary metabolites such as flavonoids to resist PEF-induced abiotic stress [40]. The results demonstrated that PEF treatment significantly promoted total phenol and total flavonoid accumulation in chickpea sprouts, and remarkably improved their DPPH and ABTS radical scavenging activities, achieving simultaneous enhancement of nutritional value and functional activity [41].

The enrichment of flavonoids may be attributed to PEF-induced activation of the phenylpropanoid metabolic pathway and its downstream flavonoid biosynthetic branch. In the upstream phenylpropanoid pathway, PEF significantly elevated the activities of key enzymes PAL, C4H, and 4CL, which sequentially catalyze the conversion of L-phenylalanine to p-coumaroyl-CoA, the universal precursor for flavonoid biosynthesis. Consistent with the enzyme activity changes, the relative expression levels of *CaC4H* and *Ca4CL* were also significantly increased, driving more carbon flux into flavonoid synthesis and ensuring adequate precursor supply [42]. With sufficient substrate availability, downstream key genes were also coordinately upregulated: *CaCHS* encodes chalcone synthase that catalyzes the first committed step of flavonoid skeleton formation, while *CaCHI1A* encodes chalcone isomerase that mediates stereospecific isomerization into the flavanone core structure. The significantly upregulated expression of both genes effectively directed precursor flux toward flavonoid biosynthesis and reduced substrate consumption via competing side branches [43]. Conclusively, PEF treatment under appropriate conditions induces mild abiotic stress via reversible electroporation, upregulates the expression of key genes in the phenylpropanoid-flavonoid pathway, and promotes the accumulation of flavonoid active substances in chickpea sprouts.

## 5. Conclusions

PEF treatment effectively enhanced flavonoid accumulation and antioxidant capacity in chickpea (cv. Pinying No. 1) sprouts during germination. The optimal conditions for the PEF treatment were: 30 kV, 500 Hz, 6 µs pulse width, 27 min, applied after 12 h of soaking, followed by 72 h of germination, under which the total flavonoid content reached 3025.27 ± 21.11 µg/g DW. PEF-treated sprouts exhibited reduced MDA and ROS levels, enhanced antioxidant enzyme activities and gene expression levels, and activated phenylpropanoid pathway enzymes and genes, accounting for the flavonoid enrichment. PEF-assisted germination is an effective non-thermal strategy for producing flavonoid-enriched chickpea sprouts.

## Figures and Tables

**Table 1:** Regression model variance analysis.

Variables	Sum of Squares	*df*	Means Square	F Value	*p* Value
Model	637,700.00	9	70,859.4	92.84	<0.0001
A-treatment time (min)	62,498.8	1	62,498.8	81.88	<0.0001
B-treatment voltage (kV)	3368.56	1	3368.56	4.41	0.0738
C-treatment frequency (Hz)	85,652.47	1	85,652.47	112.22	<0.0001
AB	33,277.06	1	33,277.06	43.6	0.0003
AC	20,206.62	1	20,206.62	26.47	0.001
BC	33,907.54	1	33,907.54	44.42	0.0003
A^2^	49,250.37	1	49,250.37	64.52	<0.0001
B^2^	117,500.00	1	117,500.00	153.91	<0.0001
C^2^	193,700.00	1	193,700.00	253.77	<0.0001
Residual	5342.98	7	763.28		
Lack of fit	2203.41	3	734.47	0.9358	0.5017
Pure Error	3139.56	4	784.89		
Corrected total	643,100.00	16			
*R*^2^ = 0.9917		Adjusted *R*^2^ = 0.9810		Predicted *R*^2^ = 0.9376	

## Data Availability

The original contributions presented in the study are included in the article/Supplementary Materials; further inquiries can be directed to the corresponding authors.

## Supplementary Materials

The following supporting information can be downloaded at: https://www.mdpi.com/article/10.3390/foods15203750/s1, Table S1: Factors and levels of response surface methodology. Table S2: Primer sequences used in the study.
