# Supplementary material for: Pulsed Electric Field Enhances Flavonoid Accumulation and Antioxidant Capacity in Chickpea Sprouts by Activating the Phenylpropanoid Pathway and Antioxidant System
Source: Foods. 2026 Oct 21;15(20):3750. doi: 10.3390/foods15203750 (PMC13704510; doi:10.3390/foods15203750)
Supplement: Supplementary file 1 [file foods-15-03750-s001.zip › foods-4607525-supplementary.pdf]

Table S1. Factors and levels of response surface methodology

Factor	Coding	Level		
		-1	0	1
A:Processing time（min）	A	20	25	30
B:Rocessing voltage (kV)	B	25	30	35
D:Processing frequency (Hz)	C	400	500	600

Table S2. Primer sequences used in the study.

Primer	Forward Primer (5'-3')	Reverse Primer (5'-3')
Actin	TTGGAGACGAGCAGCGTTTCAGA	GCCTGCTGCTTCCATTCCT
CaAPX	CGACCATGTACTGCATGGCA	TTAGTTTCCAAGTAGGGATGTCAAAA
CaC4H	CGACCATGGCATGGATCTTC	TTAATTAAATGATCTTGGCTTACAAACA
CaCHI1A	GACCATGGCATGGCCTCA	TTAAACGAATTGATCCAAATATTCATC
CaCHS	CGACCATGGCATGGCTAGTG	CAAGGCTTGTGTTGACATGG
CaHID	CGACCATGGCATGGCTTC	TCAAACAAGAAAGGCAGCAATACG
CaPAL	CGACCATGGCATGGAAGC	TTAACAAACTGGAAGAGGAGCACC
CaPOD	CGACCATGGCATGGATTTTG	TTACATTGAGCTAACCATACCCTCC
CaSOD	CGACCATGGCATGAAATTGC	CTAATCAGTATCACTATCAGCAAATATC
Ca4CL	TGATGACGTGGTGGCTCTTC	CTCTCCATCCACCTGCTGTG
